# Patterns of Multimodality Management of Gastric Cancer—Single Institutional Experience of 372 Cases From a Tertiary Care Center in North India

**DOI:** 10.3389/fonc.2022.877493

**Published:** 2022-05-02

**Authors:** Sunil Kumar, Naveen Kumar, Suryanarayana Deo, Sandeep Bhoriwal, Amitabha Mandal, Atul Sharma, Sushmita Pathy, Prasenjit Das, Sanjay Thulkar, Sushma Bhatnagar

**Affiliations:** ^1^ Department of Surgical Oncology, DR. Bhim Rao Ambedkar Institute Rotary Cancer Hospital (DRBRAIRCH), All India Institute of Medical Sciences, New Delhi, India; ^2^ Department of Medical Oncology, DR. Bhim Rao Ambedkar Institute Rotary Cancer Hospital (DRBRAIRCH), All India Institute of Medical Sciences, New Delhi, India; ^3^ Department of Radiation Oncology, DR. Bhim Rao Ambedkar Institute Rotary Cancer Hospital (DRBRAIRCH), All India Institute of Medical Sciences, New Delhi, India; ^4^ Department of Pathology, All India Institute of Medical Sciences, New Delhi, India; ^5^ Department of Oncoradiology, DR. Bhim Rao Ambedkar Institute Rotary Cancer Hospital (DRBRAIRCH), All India Institute of Medical Sciences, New Delhi, India; ^6^ Department of Oncoanasthesia, DR. Bhim Rao Ambedkar Institute Rotary Cancer Hospital (DRBRAIRCH), All India Institute of Medical Sciences, New Delhi, India

**Keywords:** gastric cancer, multimodal, outcomes, survival, India

## Abstract

**Introduction:**

Worldwide gastric cancer is the 5th most commonly diagnosed cancer and the leading cause of gastrointestinal cancer-related deaths. Alone surgery provides long-term survival improvements in 20% of the patients with local advanced gastric cancer. The results can be improved considering multimodal management including chemotherapy and radiotherapy. However, in low middle-income countries like India, multimodal management is challenging. Herein, we evaluated the experience of multimodal management of gastric cancer and the long-term outcome.

**Methods:**

Retrospective analysis of the data of 372 patients was done from a prospectively maintained computerized database from 1994 to 2021. Records were analyzed for demographic details, treatment patterns, recurrences, and long-term outcomes (DFS and OS). Statistical analysis was done with the package SPSS version 26 (IBM Corp, Chicago, Illinois, USA).

**Results:**

This study included 372 patients. The mean age of the patients was 54.07. A total of 307 patients (82.5%) were operated upfront, 45 (12%) received NACT, and 20 (5.5%) underwent the palliative procedure. A total of 53.2% underwent curative resection. R0 resection rate was achieved in 95% of patients. A total of 72.58% of patients required adjuvant treatment, and the majority of the patients underwent chemoradiotherapy. The most common site of metastasis was the liver. Median follow-up was 50.16 months. The 3-year disease-free survival and overall survival were 36.28% and 67.8%, and the 5-year disease-free survival and overall survival were 30.15% and 37.7%, respectively.

**Conclusion:**

Our study suggested that multimodal management is required in locally advanced gastric cancer to achieve good long-term outcomes. The treatment sequence can be tailored based on the available resources.

## Introduction

Worldwide gastric cancer is the 5th most commonly diagnosed cancer and ranks 3rd in cancer-related death (1). Although gastric cancer was the leading cause of cancer death till the 1980s, the incidence has declined rapidly since the last few decades in most parts of the world (2–4). The decline in gastric cancer incidence was due to identifying *Helicobacter pylori* as a risk factor and modifying dietary factors. The rate of decline of gastric cancer is more profound in the United Kingdom, whereas in a country like Japan with a very high incidence of gastric cancer, the decline rate is slower. Almost two-thirds of the gastric patients are found with advanced stage, whereas 50% of patients are detected at the early stage in East Asian countries like Japan and Korea because of the endoscopic screening program (5, 6).

Gastric cancer is a lethal disease with persistently high mortality due to its presence in the advanced stage and change in the distribution of tumor location from pylorus and antrum to body and cardia (7). Despite the aggressive nature of the disease, the prognosis of gastric cancer had improved significantly in the last two decades due to improvement in surgical management and multimodal therapy. If it is diagnosed in the early stage, very good survival outcomes can be achieved with multimodal management (8). Multimodal management includes surgical management, ranging from endoscopic mucosal resection to gastrectomy, lymph node dissection, neoadjuvant chemotherapy (NACT), perioperative chemotherapy, adjuvant chemoradiation, and adjuvant chemotherapy. Advanced stage disease has a very dismal prognosis; multimodal treatment approach may prolong the survival. This study aims to evaluate the basic demographic characteristic, multimodal approach to gastric cancer, resectability rate, the response of neoadjuvant therapy, recurrence pattern, and long-term survival of gastric cancer in a high-volume tertiary cancer care center in North India.

## Materials and Methods

Retrospective analysis of the data of 372 patients was done from a prospectively maintained computerized database from 1994 to 2021. All the gastric patients were registered in the gastrointestinal cancer clinic. Multimodal management was planned. Upper gastrointestinal endoscopy (UGIE) and biopsy were done in all patients for diagnosis and extent of intraluminal disease. Contrast-enhanced computed tomography (CECT) of the chest, abdomen, and pelvis was done for the staging of the disease.

### Treatment Protocol

Before the neoadjuvant era, upfront surgery was offered in all potentially operable cases and neoadjuvant chemotherapy was only offered to the patients who were initially unresectable or locally advanced without evidence of distant metastasis. Adjuvant therapy was given to patients with a pathological T3 or above and node-positive disease.

In the last decade, neoadjuvant chemotherapy has gained ground significantly and now it is a standard treatment in locally advanced gastric cancer after the results of several randomized trials (9, 10). We followed the same treatment protocol. In all locally advanced tumors (T3/T4 or node positive), neoadjuvant chemotherapy followed by surgery was done. Upfront surgery was performed in only emergency indications like bleeding or gastric outlet obstruction.

#### Neoadjuvant Chemotherapy Regimens

We used epirubicine, cisplatin, and 5-FU (ECF); folinic acid, fluorouracil, and oxaliplatin (FOLFOX); and capecitabine and oxaliplatine (CAPOX) before FLOT era. After the FLOT4-AIO trial, 5-fluorouracile, leucovorin, oxaliplatin, and docetaxel (FLOT) regimen was an integral part of our NACT schedule for patients with good performance status (ECOG 0 or 1), and FOLFOX for poor performance status patients (2 or 3).

#### Adjuvant Treatment

Adjuvant treatment was offered to patients with pathological T3/T4 or node-positive disease. Adjuvant chemotherapy alone was given to the patients with adequate lymph node dissection (D2 lymphadenectomy), and optimum lymph node was evaluated in histopathological examination (16 nodes). Adjuvant chemoradiation as per McDonald’s protocol (chemotherapy: fluorouracil and leucovorin; radiotherapy: 45 Gy of radiation at 1.80 Gy per day, 5 days per week for 5 weeks, with intensity-modulated radiation therapy technique) was given to those patients who had inadequate lymph node dissection (less than D2 dissection) and less than 16 nodes evaluated in the pathological examination.

### Statistical Analysis

Categorical variables were expressed in percentages and frequencies. A chi-square test was used for group comparison of categorical variables. The software package SPSS version 26 (IBM Corp, Chicago, Illinois, USA) was used for the statistical analysis. All values of *p* < 0.05 were taken as statistical significance. Disease-free survival (DFS) was calculated from the date of completion of the treatment to recurrence or death, whichever comes earlier. Overall survival (OS) was calculated from the date of registration to death or lost to follow up, whichever comes earlier. Kaplan–Meier estimate was used for survival analysis.

## Results

This study included 372 patients. The mean age of the patients was 54.07 (range 17–84 years), with male predominance. Pain in the abdomen was the most common presenting symptom followed by anorexia and weight loss. The antropyloric region was the most common site of tumor occurrence. The majority of the patients were presented with locally advanced stages (stage II and stage III). The demographic profile of the patients is shown in **Table 1**. Among all the patients, 63 (16.9%), 39 (10.5%), and 36 patients (9.7%) were smokers, alcoholics, and tobacco chewers, respectively. Most of the patients were presented with good performance status (**Table 1**).

**Table 1:** Demographic profile and baseline characteristics of the patients.

Sl N	Parameters	Variables	No. of patients (*n* = 372)	Percentage
**1**	**Sex**	Male	274	(73.3%)
		Female	98	(26.7%)
**2**	**Symptoms**	Pain	222	59.7%
		Anorexia	106	28.5%
		Weight loss	104	28%
		Dyspepsia	68	18.3%
		Vomiting	65	17.5%
		Malena	10	4.3%
		Hematemesis	6	1.9%
		GOO	81	21.8%
		Abdominal mass	54	14.5%
**3**	**Location**	Antropyloric	230	61.8%
		Body	111	29.8%
		Cardia	24	6.5%
		GE Junction	7	1.9%
**4**	**CT findings**	Perigastric node	107	28.7%
		Ascites	12	3.2%
		Omental nodule	4	1.1%
		Peritoneal deposit	1	0.3%
		Liver mets	1	0.3%
**5**	**Clinical stage**	Stage 1	17	4.6%
		Stage 2	76	20.4%
		Stage 3	217	58.3%
		Stage 4A	28	7.5%
		Stage 4B	34	9.1%
**6**	**ECOG**	0	22	5.9%
		1	270	72.6%
		2	58	15.6%
		3	22	5.9%

A total of 307 patients (82.5%) were operated upfront, 45 (12%) patients were planned for neoadjuvant chemotherapy (NACT) followed by reassessment for surgery, and 20 (5.5%) cases underwent the palliative procedure. Among NACT patients, complete response (CR) and partial response (PR) were noted in 1 (0.5%) and 24 (6.5%) respectively; 17 (4.6%) patients had stable disease and 2 (0.5%) had progressive disease. Curative resection was done in 19 patients (42.2%) after NACT, and the rest of the 26 patients were unresectable on exploration and underwent palliative surgery.

The curative resection rate in this study was 53.4% (199 patients). The most commonly performed surgical procedure was distal radical gastrectomy followed by total gastrectomy. Clavien Dindo grade 3–4 was seen in 16 (7.5%) patients. R0 resection rate was achieved in 95% of the patients. The mean node harvested was 15, ranging from 6 to 32, and the mean pathological node involvement was 3 (range 1–16). Surgical details are shown in **Table 2**. After palliative surgery, 51 (13.3%) patients did not receive any form of palliative therapy, 102 (27.4%) patients received palliative chemotherapy, 2 (0.5%) patients received palliative radiotherapy, and the remaining 18 patients (4.8%) received the best supportive care.

**Table 2:** Surgical details and pathological parameters.

Sl N	Parameters	Variables	No. of patients (*n* = 372)	Percentage
**1**	**Type of surgery**	Curative resection	199	53.4%
		Unresectable on exploration	93	25.1%
		Palliative procedure	80	21.5%
**2**	**Surgical procedure**	Distal radical gastrectomy (DRG)	119	31.6%
		Total gastrectomy	46	12.4%
		Subtotal gastrectomy	31	8.4%
		Esophago-gastrectomy	1	0.3%
		Wedge resection	2	0.5%
		Palliative procedure (Unresectable + palliative surgery)	173	46.8%
**3**	**Exploration findings**	Omental deposits	74	19.9%
		Peritoneal deposits	68	18.3%
		Liver metastasis	37	9.9%
		Colonic involvement	37	9.9%
		Pancreas involvement	37	9.9%
		Ascites	37	9.9%
		Mesenteric deposits	25	6.7%
		Duodenal involvement	29	7.8%
		Celiac axis involvement	18	4.8%
**4**	**Margin positivity**	Proximal margin	4	1.1%
		Distal margin	6	1.6%
		Final margin positivity	10	5.1%
**5**	**Pathological stage**	Stage 0	3	1.5%
		Stage 1	14	7.1%
		Stage 2	85	42.7%
		Stage 3	90	45.2%
		Stage 4	7	3.5%

After curative resection, 143 (72.58%) patients received adjuvant therapy, and 54 (27.4%) did not receive any adjuvant therapy. Out of 143 patients, 30 (15.2%) patients received adjuvant chemotherapy and 103 (52.3%) patients received adjuvant chemoradiotherapy. Palliative RT and palliative chemotherapy were offered to 3 (1.5%) patients each, whereas 1 patient went for the best supportive therapy; the remaining 18 patients (9.13%) lost their follow-up. The majority of the patients developed systemic recurrence, and the liver was the most common site of systemic recurrence. Systemic, local, and locoregional recurrence occurred in 72 patients (36.2%), 16 patients (8%), and 13 patients (6.5%), respectively. Among all systemic recurrences, 33 (16.6%) patients had liver metastasis followed by peritoneum in 29 (14.6%) patients.

**Figure 1:**
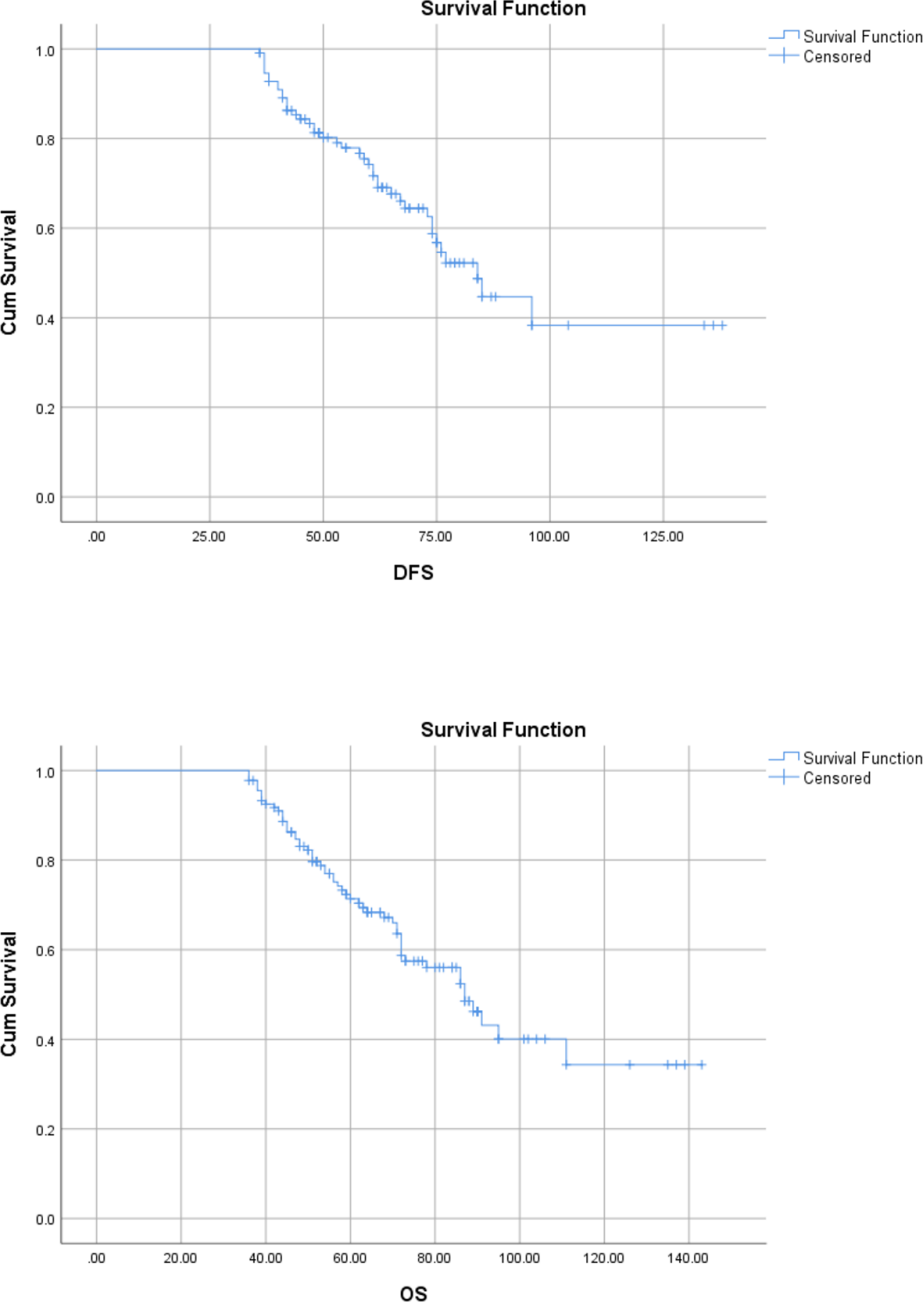
Kaplan–Meier curve showing 3-year disease-free and overall survival.

**Figure 2:**
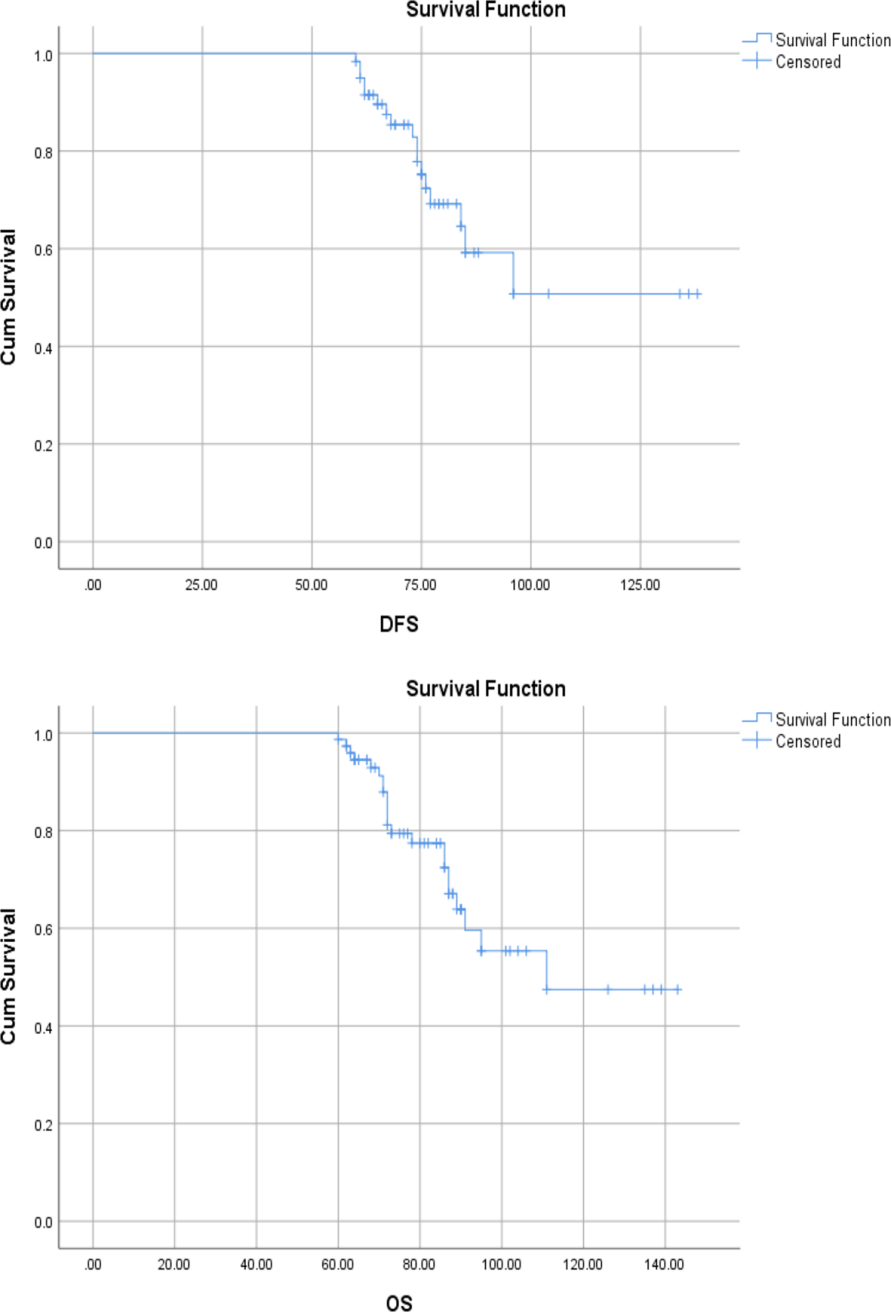
Kaplan–Meier curve showing 5-year disease-free and overall survival.

Survival analysis was done for only those patients who underwent curative resection. The median follow-up was 50.166 months. The 3-year DFS and OS were 36.28% and 67.8%, respectively. The 3-year median DFS and OS were 61 (95% CI, 46.6–75.3) and 84 (95% CI, 85.1–103.1) months (**Figure 1**). The 5-year DFS and OS were 30.15% and 37.7%, respectively, and the 5-year median DFS and OS were 63 months (95% CI, 96.2–119.7) and (95% CI, 52.7–73.2) (**Figure 2**).

## Discussion

Although the incidence of gastric cancer has declined in most countries, it is still a major cause of cancer-related mortality (2, 7). The lethality of gastric cancer lies in its presentation in late stage, due to vague symptomatology. Until or unless there are features of gastric outlet obstruction, hematemesis, abdominal lump, or gross weight loss, the disease does not attract the attention of the patients (11, 12). In this study, the most common symptom was pain in the abdomen. Worldwide, the incidence of stomach cancer has declined significantly, mainly in European countries, due to modification in dietary factors and identification of *H. pylori* as a risk factor. Other risk factors are smoking, alcohol intake, and tobacco chewing (13). In our study, almost 35% of the patients were associated with predisposing factors.

In the last two decades, the trends of the location of the tumor in the stomach have shifted more proximally from the location in the distal stomach (14). In the current study, the distal stomach is the most common site of cancer.

Traditionally, the staging of gastric cancer is done with a CT scan; however, various studies have been conducted to see the role of 18-FDG PET scan. PET scan has certain limitations in diagnosing gastric cancer because the normal gastric mucosa and benign lesions take FDG uptake and are difficult to differentiate with pathological uptake. Few studies have reported a limited role in stage IV disease (peritoneal carcinomatosis) with low sensitivity (range: 9%–50%; median: 32.5%) and marginal higher specificity (63%–99%; median: 88.5%) (15). Thus, the role of PET scan is still evolving. At our center, a PET scan is advised only in recurrent or stage IV cases to prognosticate the disease.

The sequence of choice of the multimodal treatment depends on various factors like patient’s performance status (ECOG performance status), comorbidity, and site and stage of the disease. Treatment options for early gastric cancer are eradication of *H. pylori*, endoscopic therapy, gastrectomy, and adjuvant therapy (16). In developing countries, endoscopic resection expertise is still lesser, and gastrectomy is commonly preferred. In our study, only 4.6% of the patients presented with early gastric cancer and all patients underwent gastrectomies.

Locally advanced gastric cancer requires multimodal management. Many randomized trials and one meta-analysis proved that using NACT or perioperative chemotherapy has a survival advantage over upfront surgery for potentially resectable gastric cancer (9, 17). In our study, more than 50% of patients had a locally advanced stage and required multimodal management. The resectability rate after NACT was 42.2%, which is a bit lower than other studies. The pathological CR rate is lower (0.5%) in this study as compared to literature (5%–15%) (18, 19). This difference was seen because of the advanced stage at the presentation.

Gastrectomy with adequate lymph node dissection (at least 16 nodes) is the surgical procedure of choice for operable gastric cancer with good quality of life. Proximal tumors involving cardia, fundus, and GE junction are treated with total gastrectomy and distal tumors (body, antropyloric area) with subtotal or distal gastrectomy. In our series, most of the patients underwent distal radical gastrectomy since the majority of the patients had distal gastric cancer and total gastrectomy was only performed for proximal tumors or involvement of the whole stomach. In the literature, two major trials compared subtotal with total gastrectomy for distal gastric cancer without any significant survival advantage in favor of total gastrectomy (20, 21). In this study, distant metastasis, involvement of celiac axis, hepatic artery, and aorta were considered as unresectable diseases, and require palliative surgery (56%) in the form of feeding jejunostomy or gastrojejunostomy.

The extent of lymphadenectomy is an area of active debate for a long time. Various types of lymph node dissection is described in the literature, D1 (Station 1-6), D1+ (Station 1-6, and 8a, 9, 11), D2 (Station 1-12a), and D3 (Station 1-16 or D2+ paraaortic node dissection). Japanese and Korean surgeons preferred more aggressive lymph node dissection, whereas according to current NCCN guidelines, spleen and pancreas preserving D2 lymphadenectomy with at least 15 nodes for histopathological examination is the standard of care (22–24). In the current study, all the patients underwent D2 lymph node dissection and the complication rate is comparable. Most of the prospective randomized trials have failed to demonstrate the survival advantage of D2 over D1 lymphadenectomy. The two largest prospective randomized trials (MRC, Dutch), which are debated the most, also did not find any significant survival advantage of D2 over D1 lymphadenectomy; however, long-term analysis of these studies had shown disease-specific survival benefits (25–28).

Targeted therapy such as trastuzumab has been established for unresectable and metastatic HER2 positive gastric cancer. Many trials like ToGA, LOGiC, and TyTNHA showed an improvement in survival after using the HER2-targeted therapy. Various phase III trials are ongoing to explore other targeted therapies based on epidermal growth factor receptor (EGFR), vascular endothelial growth factor (VEGF)/vascular endothelial growth factor receptor (VEGFR), MET, or the mechanistic target of rapamycin (mTOR) (29).

Systemic and locoregional recurrence are the two forms of recurrence in gastric cancer, where systemic is common. In one study, systemic and locoregional recurrences were 60% and 40%, respectively (30). Another study quoted locoregional recurrence rate in 15% of cases; peritoneal, 49%; nonperitoneal distant recurrence, 54%; and liver metastasis, 20% (31). Another study evaluated the recurrence pattern in proximal gastric cancer and found a recurrence rate of 85.9% within 2 years, where locoregional recurrence was the most common pattern followed by hematogenous. Among them, liver was the most common organ for systemic recurrence followed by the peritoneum (32). In this current study, systemic recurrence (36.2%) was the most common form of relapse and liver was the most common site.

Survival after curative resection depends on stage, location, and ethnicity. The Asian population has better survival than the Western population (32). In the literature, 5-year survival of locally advanced gastric cancer is reported with a range of 40%–60% after multimodal management (25, 26, 33). Our study has shown almost similar outcomes.

## Conclusion

Although the incidence of gastric cancer has been decreased in the Indian population, it is still a deadly disease, because of its aggressive biology and late presentation. Our study suggested that optimal outcomes of gastric cancer in low middle-income countries can be achieved based on the best available resources using a multimodal treatment approach.

## Data Availability Statement

The original contributions presented in the study are included in the article/supplementary material. Further inquiries can be directed to the corresponding author.

## Ethics Statement

Ethical review and approval were not required for the study on human participants in accordance with the local legislation and institutional requirements. Written informed consent for participation was not required for this study in accordance with the national legislation and the institutional requirements.

## Author Contributions

SD started the surgical program and provided extensive guidance in making the manuscript. NK and AM analyzed the data and wrote the manuscript. SK and SBho reviewed the manuscript and added their inputs. AS were the lead medical oncologist and added the inputs. SP was a lead radiation oncologist and PD was the lead pathologist and they added their inputs. All authors contributed to the article and approved the submitted version.

## Conflict of Interest

The authors declare that the research was conducted in the absence of any commercial or financial relationships that could be construed as a potential conflict of interest.

## Publisher’s Note

All claims expressed in this article are solely those of the authors and do not necessarily represent those of their affiliated organizations, or those of the publisher, the editors and the reviewers. Any product that may be evaluated in this article, or claim that may be made by its manufacturer, is not guaranteed or endorsed by the publisher.
